# A biobehavioral observational study to understand the multilevel determinants of cardiovascular health in Black women: the BLOOM Study protocol

**DOI:** 10.1186/s12905-024-03182-0

**Published:** 2024-07-05

**Authors:** Yue Liao, R. Matthew Brothers, Kyrah K. Brown, Rebecca E. Lee

**Affiliations:** 1 Department of Kinesiology, College of Nursing and Health Innovation, The University of Texas at Arlington, Arlington, TX USA; 2 Center for Health Promotion and Disease Prevention, Edson College of Nursing and Health Innovation, Arizona State University, Phoenix, AZ USA

**Keywords:** Women’s health, Lifestyle behaviors, Racial discrimination, Microaggression, Remote monitoring, Ambulatory assessment, Experience sampling, Social determinants of health, Vascular health, Cardiovascular disease

## Abstract

**Background:**

The racial/ethnic and gender disparities in cardiovascular disease (CVD) morbidity and mortality in the United States are evident. Across nearly every metric, non-Hispanic Black women have poorer overall cardiovascular health. Emerging evidence shows a disproportionately high burden of increased CVD risk factors in Black women of childbearing age, which has a far-reaching impact on both maternal and child outcomes, resulting in premature onset of CVD and further widens the racial disparities in CVD. There is growing recognition that the fundamental driver of persistent racial/ethnic disparities in CVD, as well as disparities in behavioral risk factors such as physical activity and sleep, is structural racism. Further, the lived personal experience of racial discrimination not only has a negative impact on health behaviors, but also links to various physiological pathways to CVD risks, such as internalized stress resulting in a pro-inflammatory state. Limited research, however, has examined the interaction between daily experience and health behaviors, which are influenced by upstream social determinants of health, and the downstream effect on biological/physiological indicators of cardiovascular health in non-pregnant Black women of childbearing age.

**Methods/Design:**

The BLOOM Study is an observational study that combines real-time ambulatory assessments over a 10-day monitoring period with in-depth cross-sectional lab-based physiological and biological assessments. We will use a wrist-worn actigraphy device to capture 24-h movement behaviors and electronic ecological momentary assessment to capture perceived discrimination, microaggression, and stress. Blood pressure will be captured continuously through a wristband. Saliva samples will be self-collected to assess cortisol level as a biomarker of psychological stress. Lab assessments include a fasting venous blood sample, and assessment of various indices of peripheral and cerebral vascular function/health. Participants’ address or primary residence will be used to obtain neighborhood-level built environmental and social environmental characteristics. We plan to enroll 80 healthy Black women who are between 18 and 49 years old for this study.

**Discussion:**

Results from this study will inform the development of multilevel (i.e., individual, interpersonal, and social-environmental levels) lifestyle interventions tailored to Black women based on their lived experiences with the goal of reducing CVD risk.

**ClinicalTrials.gov Identifier:**

NCT06150989.

**Supplementary Information:**

The online version contains supplementary material available at 10.1186/s12905-024-03182-0.

## Introduction

In the United States, the prevalence of CVD risk factors (e.g., hypertension and diabetes mellitus) among women of childbearing age (18–49 years) has increased substantially over the last two decades [1]. Compared to non-Hispanic White women, non-Hispanic Black women (hereafter referred to as Black women) are more likely to have comorbid hypertension and diabetes, and more likely to have these comorbidities undiagnosed [2]. In addition to traditional CVD risk factors, adverse health behaviors, such as physical inactivity, insufficient sleep and poor-quality sleep, are recognized as modifiable factors that contribute to CVD disparities in Black Americans [3]. Compared to other racial/ethnic groups, Black women are less likely to report getting the recommended levels of physical activity [4], and report a higher prevalence of shorter sleep, having trouble falling asleep, trouble staying asleep, and not waking up most days feeling rested (≥ 4 in the previous week) [5]. There is an increasing interest in investigating the combined effects of 24-h movement behaviors on health [6]. From a movement perspective, the 24-h period is distributed among sleep, sedentary behavior, and physical activity. The time reallocation between sleep, sedentary behavior, and physical activity is associated with a number of health outcomes, such as physiologically relevant reductions in mortality risk and negative cardiometabolic biomarkers [7]. Thus, from a lifestyle intervention perspective, it is imperative to consider and target the three 24-h movement behaviors to optimally decrease CVD risk and maximize health benefits. The 24-hr movement guidelines outline recommendations for physical activity, sedentary behavior, and sleep based on empirical evidence [8]. Emerging evidence shows that this guideline adherence was lower in females than males; and lowest among non-Hispanic Blacks compared to other racial groups [9]. With these consideration in mind, this study is focused on physical activity, sedentary behavior, and sleep as key daily modifiable health behaviors.

It is widely recognized that the fundamental driver of persistent racial/ethnic disparities in CVD is structural racism [10]. Structural racism refers to the totality of ways in which society fosters racial discrimination through mutually reinforcing systems of housing, education, employment, healthcare, and other social determinants of health (SDOH) [10, 11]. Structural racism creates stressful and unhealthy social and built environments, which contributes to disparate behavioral risk factors such as physical inactivity and inadequate sleep among Black women [10, 12]. Several social and structural factors are associated with lower levels of physical activity among Black women [13], such as residential racial segregation resulting in communities with fewer physical characteristics that promote physical activity [14, 15], perceived time availability and monetary cost of exercise facilities, family/caregiving responsibilities, lack of physical activity partner or role models [16]. Similarly, decades of sleep disparities research indicates that several neighborhood and environmental factors, such as residential racial segregation (which can transcend socioeconomic status), and neighborhood ambient features of urban environments, such as loud noises or bright lights, can be deleterious to sleep [17].

In addition to impacting the social and built environments, systematic inequality also creates social stress from experience of racial discrimination and microaggressions. This type of social stress has been associated with unhealthy behaviors and may provoke severe psychological and physiological responses that lead to poor health outcomes [18–20]. For example, self-reported experiences of racial discrimination have been associated with shorter sleep duration [21] and increased sedentary time [22] among Black women. There are several known physiological pathways that link racism and racial discrimination to CVD risks, such as abnormal activation of the sympathetic nervous system from internalized stress, resulting in the augmented release of cortisol [23]. Previous studies have also shown an association between perceived discrimination and inflammatory markers such as high C-reactive protein (CRP) and interleukin-6 (IL-6) levels [20, 24]. Ultimately, constant psychological stress from adverse social conditions can cause biological responses (i.e., allostasis), which, over time, can cause neurohormonal imbalances, including augmented systemic inflammation and oxidative stress (i.e., allostatic overload) [25, 26]. These alterations can subsequently impair vascular function and responsiveness [27–29], which is an early indicator of atherogenesis and CVD risk [30].

**Fig. 1:**
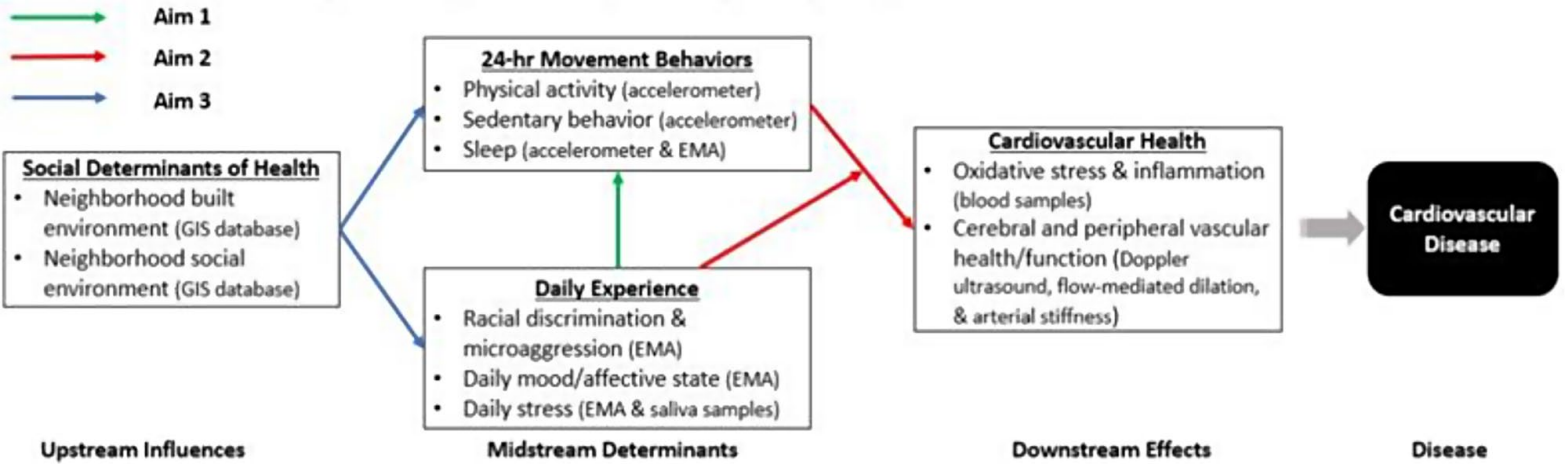
Conceptual diagram by study aims and measures

Overall, previous work has shown evidence of how neighborhood-level characteristics could impact individual behaviors and suggested a link between social stress and CVD risk. However, limited studies have focused on how an individual’s *actual experiences and behaviors in their everyday life* may contribute to validated, measurable, and quantifiable biomarkers of CVD risk. This knowledge gap is particularly critical for effective lifestyle intervention development, because in order to promote sustainable behavioral change, a lifestyle intervention will need to consider the everyday contexts of a person’s life in the real-world setting. Accordingly, this observational study aims to: (1) Examine the impact of daily experience (i.e., racial discrimination, affective states, stress) on health behaviors (i.e., physical activity, sedentary behavior, sleep) at the intrapersonal level among Black women. We hypothesize that there is an acute positive association between negative daily experiences and unhealthy behaviors, i.e., a higher level of stress will lead to more time spent in sedentary behaviors that day and poorer sleep at night. (2) Test the association between daily behaviors and impairments in biomarkers associated with vascular function/health (i.e., augmented systemic inflammation and oxidative stress, impaired peripheral/cerebral vascular function, increased large artery stiffness), as well as the impact of daily experience on the relationship between behaviors and vascular function. We hypothesize that unhealthy behaviors are positively associated with vascular function impairments, and daily experiences moderate this association, i.e., negative daily experiences will augment the negative impact of unhealthy behaviors on vascular function. (3) Explore the influence of neighborhood-level characteristics (i.e., social environment factors: i.e., neighborhood income and poverty, racial composition; and built environment context, such as park density and walkability) on daily experience and health behaviors. We hypothesize that unfavorable neighborhood characteristics are associated with unhealthy daily behaviors and negative daily experiences. Figure 1 outlines the study’s conceptual diagram.

## Methods

### Participants recruitment and eligibility

Black women (*n* = 80) will be recruited from the Dallas-Fort Worth Metroplex (Black residents make the 3rd largest racial group in this area). The research team will collaborate with local organizations, including the Tarrant County Health Equity Alliance [31], to assist with recruitment efforts. Study information will be shared at community events, newsletters, and email listservs. The research team will also apply strategies previously identified by scholars with expertise in recruitment and collaboration with Black women [32–34]. Participants can also be recruited from the University of Texas at Arlington (UTA) campus (e.g., students and staff) and through focused social media advertisement (e.g., filtered by geographic location, age, gender).

Interested women will complete a brief online screener to determine eligibility. To be eligible, participants must be assigned female at birth; between 18 and 49 years old; self-identify as Black or African American; not currently pregnant; ownership of a smartphone; and able to speak and read English. *Exclusion criteria* include diagnosed hypertension, cardiovascular, respiratory, metabolic, and/or neurological disorders; functional limitations or health issues that preclude physical activity; currently taking medications for thyroid function or psychological conditions such as depression, anxiety, and mood disorders; current use of oral or inhalant corticosteroids for asthma; or have regularly smoked within the last 2 years.

Recruitment for the study started in February 2024. The first study participant was enrolled on March 4th, 2024. As of May 2024, a total of 63 women completed the eligibility screener, and 27 of them were eligible for the study. Among women who were not eligible for the study, main reason was their current medication use (*n* = 15), followed by medical conditions (*n* = 12) and smoking status (*n* = 4). This study is currently enrolling participants, and we expect to finish study enrollment around October 2025.

### Study procedures

This cross-sectional observational study, **B**lack women’s **L**ife experience **O**n cardiovascular health via **O**ngoing **M**onitoring (BLOOM), will involve two in-person visits at UTA with a 10-day ambulatory monitoring at participants’ free-living environments. The primary purpose of the first visit is to conduct the lab-based physiological assessments (i.e., a fasted blood draw and peripheral & cerebral vascular function/health) and provide the ambulatory assessment tools and associated instructions to participants (i.e., a wrist-worn accelerometer, a wrist-worn blood pressure monitor, a smartphone application [app], and a saliva self-collection kit). The study principal investigator is Dr. Liao, Yue from UTA. Drs. R. Matthew Brothers from UTA and Rebecca E. Lee from Arizona State University are the study co-investigators. Dr. Kyrah K. Brown from UTA, a board member of the Tarrant County Health Equity Alliance, served as the subject matter expert during the first year of the study (to assist with study protocol design based on her expertise and experience working and interacting with the community members). Our study protocol followed the Standard Protocol Items for Observational Studies (SPIROS) [35] guideline (see appendix). The first version of this study protocol was reviewed and approved by the UTA’s Institutional Review Board (protocol #: 2023 − 0344) on September 27, 2023. Continuing review will be performed by the IRB annually. Any serious adverse events and unanticipated problems will be reported to this institutional IRB. Amendments to the protocol will be reviewed and approved by the IRB before implementation. Because this is an observational study that involves minimal risks, a data monitoring committee is not needed. All study staff will be trained by the investigator team prior to conducting any study-related activities. We keep a detailed standard of operating procedure document that covers all aspects of study-related activities, from participant recruitment to data collection and management, to ensure study quality and consistency. This study is registered on ClinicalTrials.gov (NCT06150989, registration date: November 30, 2023).

Eligible participants will be scheduled for an in-person visit at the Physical Activity and Wearable Sensors (PAWS) Research Laboratory, located at the UTA campus in Arlington, Texas. At this in-person visit, a study staff will first provide a throughout overview of the study purposes, all study-related procedures and the potential related risks, and study compensation plan. Since this study will involve collection of blood samples from Black women, we followed the recommended guidelines to ensure a transparent process and trustworthy discussion between the research staff and study participants [36]. After this study overview and allowing participants to ask any questions they might have, participants will provide a written informed consent by signing an informed consent document for their enrollment in the study. The ambulatory monitoring period will start on the first day following the lab-based assessments (i.e., Visit #1) and will last a total of 10 days. Participants will be instructed to follow their usual behaviors and routines during this period. They will wear the accelerometer (ActiGraph wGT3X-BT) on their non-dominant wrist, wear the blood pressure wristband (Aktiia SA) on their dominant wrist, and answer 6 brief electronic surveys each day via a smartphone app. They will provide four saliva samples a day for 3 consecutive days out of the 10-day period.

At the end of the 10-day monitoring period, participants will return the study devices and all collected saliva samples. They will also complete a brief exit survey to share their opinions and feedback about this study. To encourage study procedure adherence, participants will be compensated for their time and effort on a pro-rated basis. In total, participants will receive up to $150 for completing all study procedures. Participants can stop taking part in the study at any point during the study period. In that scenario, participants will be compensated based on the study components they have completed. All study data will be kept confidential. The participants’ full names or personally identifiable information will not be requested on any study materials. Each participant will be assigned a study ID that links their data from online surveys, ambulatory monitoring devices, lab assessments, and biomarker assay data.

### Lab-based assessments (procedures and measures)

The following assessments of cardiovascular health indicators will take place during participants’ in-person study visit.

A fasting venous blood sample will be taken for assessment of a comprehensive metabolic panel (e.g., glucose, glycated hemoglobin, blood lipid profile, etc.), as well as biomarkers associated with CVD risk [37], including markers of inflammation (e.g., CRP & vascular cellular adhesion molecule-1 [VCAM-1]), oxidative stress (e.g., thiobarbituric acid reactive substances [TBARS] & superoxide), and antioxidant status. Individual assays will be performed via enzyme-linked immunosorbent assay kits. Reactive oxygen species will be measured using electron paramagnetic resonance spectroscopy and superoxide fluorescence via peripheral blood mononuclear cells as previously described [38].

*Cerebral vascular function/health* will be measured utilizing trans-cranial Doppler ultrasound assessment of blood velocity in the middle cerebral artery (MCAv_mean_) and Doppler ultrasound measures of blood flow in the extracranial arteries (e.g., internal carotid artery [ICA]) during a hypercapnic challenge induced by breathing 6% Carbon dioxide. Cerebral function/health will be defined as the % increase and slope of the response in MCAv_mean_, ICA_flow_, and ICA or MCA conductance from baseline during hypercapnia. This methodological approach is routinely performed in our laboratory [39, 40]. Importantly, this is predictive of and a contributor to various neurocognitive conditions and cerebral vascular diseases [41, 42].

*Peripheral vascular function/health* will be determined by several commonly utilized approaches. Macrovascular function will be assessed as brachial artery vasodilation following a period of suprasystolic cuff occlusion (i.e., flow-mediated vasodilation). Microvascular function will be assessed as the % change in blood velocity from baseline following cuff release. Large elastic artery health will be assessed as carotid artery intima-media thickness as well as central and peripheral artery ß-stiffness, arterial compliance, and pulse wave velocity utilizing Doppler ultrasound (GE P5) and applanation tonometry (SphygmoCor® CPV Pulse Wave Velocity System). This is critical as impairments in these variables contribute to cerebral vascular dysfunction as well as other forms of CVD [43, 44].

### Ambulatory assessments (procedures and measures)

*The 24-h Movement Behaviors* We will use the ActiGraph wGT3X-BT device to obtain measures of physical activity, sedentary behavior, and sleep. This device is a water-resistant, triaxial accelerometry that is used widely to capture continuous, high-resolution movement behaviors. Participants will wear the ActiGraph device on their non-dominant wrist at all times for 10 days. Collected data will be stored in the ActiGraph device’s internal memory and will be downloaded upon device returned at visit 2. We will apply the cut-points developed for wrist-worn ActiGraph accelerometer to define light-intensity activity, moderate-to-vigorous intensity activity, and sedentary behavior. We will follow the standard protocol to obtain sleep estimates from ActiGraph, which includes measures of sleep onset latency (SOL), time spent awake after sleep onset (WASO), and total sleep time (TST). In addition, we will use the daily electronic survey to capture self-reported sleep information to complement the ActiGraph-measured sleep data (e.g., to adjust sleep-awake time detected by ActiGraph as needed and obtain more information about perceived sleep quality). In the first daily electronic survey, adapted items from the Pittsburgh Sleep Diary will be included to capture self-reported sleep duration, sleep efficiency, and sleep quality [45].

*Daily Experience* We will use mobile-based ecological momentary assessment (EMA) to capture daily experiences. EMA is a brief survey designed to repeatedly assess individuals’ daily events, experience, and contexts multiple times a day to obtain “snapshots” of their everyday lives [46]. The EMA survey will be administered via a smartphone app, mEMA (illumivu). Illumivu is a commercially available EMA platform widely used by behavioral science researchers. EMA surveys will be prompted up to 6 times, at random intervals throughout the day, for 10 days. The random prompt window will be calculated by dividing the waking period (i.e., from participant’s typical wake time to typical bedtime) by 6, and one EMA survey will be prompted/delivered randomly during each time window. All EMA survey items to be used have been carefully selected from relevant previous studies. To capture *perceived racial discrimination and microaggression*, we will use adapted items from the Experiences of Discrimination (EOD) [47] and Gendered Racial Microaggressions Scale (GRMS) [48]. As in previous EMA studies [49], items for *mood/affective states* are chosen based on the two fundamental dimensions of affect suggested by the circumplex model (i.e., positive and negative affect). *Perceived stress* will be assessed using two items from the Perceived Stress Scale [50]. Each EMA survey will take no more than 3–5 min to complete. Responses to the EMA surveys will be automatically uploaded to the illumivu’s cloud-based server, and will be download for data analysis upon data collection completion.

*Salivary Cortisol* Saliva samples will be collected four times a day (i.e., at wakeup, 30 min after waking, before dinner, and right before bedtime) for 3 consecutive days (2 weekdays and 1 weekend days) to capture diurnal patterns of stress across the day. We will follow the collection procedures that have been used in previous studies that demonstrated high feasibility in women [51, 52]. We will use the Salivette device (Sarstedt, Inc.), a small, cotton dental sponge swab. Participants will be asked to place the swab in their mouth for 2 min and to store the samples in their home freezer as soon as possible after each collection. At the end of the 10-day study period, we will retrieve all saliva tubes from each participant and store them at a -80ºC freezer until they are assayed in batches.

*Continuous Blood Pressure* To obtain additional insights regarding participants’ daily blood pressure pattern, we will use a wrist-worn continuous blood pressure device (Aktiia) to supplement our lab-based assessments. The Aktiia bracelet automatically tracks blood pressure throughout the day and has been clinically validated for accuracy [53, 54]. It is a wearable CE-marked Class IIa medical device that records optical signals at the wrist and transforms them into the blood pressure values using pulse wave analysis algorithms after an initial calibration procedure based on a series of oscillometric measurement performed by an upper arm cuff [55]. The Aktiia bracelet can provide up to 12 blood pressure readings each day. All data will be uploaded wirelessly through an Aktiia smartphone application to a research portal specific for this study.

### Other measures

*Neighborhood environment (SDOH)* We will use the geographic information system tool, PolicyMap, to extract neighborhood-level built environment and social environment characteristics based on participants’ home addresses. We will extract neighborhood-level (e.g., at zip code-level or census track-level) social environment characteristics, including demographic indicators such as racial composition, diversity index (i.e., probability that two individuals chosen at random would be of different races/ethnicities), segregation (Theil index); neighborhood income and poverty, education attainment, employment and unemployment, and crime rate. We will also extract built environment characteristics such as park density, supermarket density, and fast-food restaurant density.

*Covariates* We will ask participants to complete a one-time online survey to assess their demographic information (e.g., age, education level, marital status, employment status, and household income), health literacy, past exposures to lifetime discrimination, and usual diet pattern. These variables will be used as covariates in our statistical models, as needed, and will be helpful in comparing our findings with previous studies and other larger population cohorts.

### Statistical considerations

Multilevel linear mixed-effect models will be used to account for repeated measures (for daily behaviors and daily experience) within an individual. We will separate the between-person and within-person effects by partitioning the variance. **Aim 1**: The daily experience predictors include (1) racial discrimination (i.e., daily EOD and GRMS score), (2) mood/affective states (i.e., daily average of positive and negative affect), (3) daily stress (i.e., daily average of perceived stress and diurnal cortisol rhythm such as total cortisol output, cortisol awakening response, diurnal cortisol slope). The daily behavior outcome variables include total daily moderate-to-vigorous physical activity minutes, total sedentary minutes, and sleep variables (i.e., SOL, WASO, TST). We will link the predictor and outcome at the day-level so that the acute impact of daily experience on behaviors can be examined. **Aim 2**: Outcome variables are indicators of cardiovascular health from lab-based measurements. We will first test the direct independent effects of daily behaviors on cardiovascular health outcomes using the mixed-effect models. Next, we will add the single-level and cross-level interaction terms to each multilevel mixed-effect model to test the moderation effects of daily experience. **Aim 3**: We will test the direct independent effects of neighborhood-level characteristics (predictors) on daily behaviors and daily experience (outcome variables) using the mixed-effect models. All tests of the hypotheses will be two-sided and an alpha level of 0.05. All analyses will be performed using SAS 9.4.

*Sample size and power* Statistical power for evaluation of this study’s objectives was calculated using the macro by Westfall et al. that achieves power estimations based on linear mixed models [56]. Power calculations were computed based on a “fully-crossed” repeated measures design with a two-sided alpha of 0.05. This study will enroll 80 participants. Table 1 displays the statistical power based on possible effect sizes based on adjusted Cohen’s *d* values. Although pilot studies are commonly underpowered, adequate power will be achieved if our study effect is greater than *d* = 0.30. This estimate is indicative of a high likelihood of the planned sample size’s statistical power. We anticipate minimal missing data in the study. However, in the unlikely case of considerable missing data, we will conduct sensitivity analysis to account for different missing data mechanisms (missing at random or missing not at random), using appropriate approaches, such as multiple imputation, pattern-mixture, or selection models [57].

**Table 1:** Effect sizes and statistical power yields for *n* = 80

Effect Size		Small			Moderate
Cohen’s d*	0.10	0.20	0.30	0.40	0.50
Statistical Power (1-*b)*	0.17	0.51	0.84	0.98	0.99

*adjusted for repeated and correlated observations

## Discussion

Our long-term research goal is to develop multilevel (i.e., addressing factors at the individual, interpersonal, and social-environmental levels) behaviorally grounded lifestyle interventions to reduce CVD risk, improve the overall health of Black women, and minimize the gaps in racial/ethnic and gender disparities in CVD. To achieve this goal, we will first examine if and how Black women’s daily health behaviors and experiences are influenced by their social and neighborhood environments, and their impact on physiological indicators of cardiovascular health. This study is guided by the SDOH framework, which addresses racial/ethnic disparities in CVD [18]. This study uniquely encompasses determinants of cardiovascular health from multiple levels with a focus on a population that is disproportionately affected by CVD outcomes.

We expect results from our study will offer critical new insights into the risk and protective behavioral factors of cardiovascular health in Black women. We will disseminate the study results to the scientific community (e.g., via scientific conference presentations and peer-review publications) as well as community members who might be interested in this topic (e.g., via our ongoing collaboration with the Tarrant County Health Equity Alliance). Scientific manuscript authorship eligibility will follow the International Committee of Medical Journal Editors’ recommendations [58]. There is a growing recognition that racial/ethnic group data disaggregation is necessary to ensure health equity in racially/ethnically minoritized populations [59]. In other words, identifying behavioral patterns (and factors influencing these behavioral patterns) and environmental context that may explain the within-group differences in cardiovascular health among Black women can provide crucial knowledge to ensure the development of more tailored and effective lifestyle interventions for this population.

### Electronic supplementary material

Below is the link to the electronic supplementary material.

Supplementary Material 1

## Data Availability

Data sharing is not applicable to this article as no datasets were generated or analysed during the current study.

## Abbreviations

BLOOM: Black women’s Life experience On cardiovascular health via Ongoing Monitoring
CVD: Cardiovascular disease
SDOH: Social determinants of health
CRP: C-reactive protein
IL-6: Interleukin-6
UTA: University of Texas at Arlington
SPIROS: Standard Protocol Items for Observational Studies
IRB: Institutional Review Board
MCA: Middle cerebral artery
ICA: Internal carotid artery
SOL: Sleep onset latency
WASO: Time spent awake after sleep onset
TST: Total sleep time
EMA: Ecological momentary assessment
EOD: Experiences of Discrimination
GRMS: Gendered Racial Microaggressions Scale
